# Case report: two cases of rhabdomyolysis following esketamine treatment

**DOI:** 10.3389/fpsyt.2024.1450092

**Published:** 2024-07-31

**Authors:** René Zeiss, Melissa Schweizer, Bernhard Connemann, Kathrin Malejko

**Affiliations:** Department of Psychiatry and Psychotherapy III, Ulm University Hospital, Ulm, Germany

**Keywords:** esketamine, rhabdomyolysis, treatment-resistant depression, case report, adverse drug reaction, ADR

## Abstract

Major depressive disorder is a mental disorder affecting millions of people worldwide. A considerable proportion of patients demonstrate a lack of response to conventional treatment. With the recent introduction of esketamine, a new treatment option has been approved for treatment-resistant depression. Although the medication is efficacious in a substantial portion of cases, rare, but possibly serious, adverse effects may occur. This case series shows two cases of rhabdomyolysis, a destruction of muscle tissue with elevated creatine kinase levels, after administration of esketamine. The first case presented is about a 33 year old male patient who suffered from a severe episode of a depressive disorder. He got nasal esketamine as an emergency treatment. While there was an initial improvement regarding the depressive symptoms, the patient developed muscle pain and fatigue after the administration of the fourth dose, with creatine kinase (CK) levels above 22,000 U/L, indicating rhabdomyolysis. Following the discontinuation of esketamine and the implementation of supportive care, the CK levels returned to normal and the depressive symptoms abated. The second case is about a 22-year-old male patient who also suffered from a severe depressive episode and got eketamine as an emergency treatment. Following the tenth dose, the patient exhibited muscle weakness and elevated CK levels (8,032 U/L), which persisted even after dose reduction. Esketamine administration was stopped, and the following monitoring demonstrated a slow return to normal levels of CK and liver enzymes. In both cases, there was no known medical history and both patients developed rhabdomyolysis after administration of esketamine. The temporal connection suggests a possible causal relationship. We found no literature on esketamine-induced rhabdomyolysis following the administration of nasal esketamine. However, these two cases emphasize the need of monitoring for laboratory changes like elevated CK-levels in patients receiving esketamine, especially considering its growing use in treatment-resistant depression.

## Background

Major depressive disorder (MDD) is a highly prevalent psychiatric disorder affecting millions of people. MDD is a significant contributor to the number of years of life lost and to economic losses because of ill health (1). MDD has an estimated lifetime prevalence of at least 10% and there is an increased risk of suicide in people suffering from major depression, which can be up to 20-fold higher (2). Although there are a number of effective treatments for depressive disorders, including psychopharmacology, various psychotherapeutic approaches and neurostimulation, a significant proportion of patients remain difficult to treat (3). After first-line treatment with antidepressants, up to 68% do not achieve remission, and up to 30% do not respond to two adequate trials of pharmacotherapy (3). Treatment with classical antidepressants also has the disadvantage that it often takes two weeks or even longer to achieve a therapeutic response (4). There is clearly a need for new, effective therapeutic approaches to the treatment of depression. One promising approach is the nasal use of esketamine, which was approved in 2018 in the US for “the treatment of depression in adults who have tried other antidepressant medicines but have not benefited from them (treatment-resistant depression)” and in 2020 by the FDA “to Treat Depressive Symptoms in Adults with Major Depressive Disorder with Acute Suicidal Ideation or Behavior” (5). By the EMA marketing authorization was issued in 2019 (6). Esketamine is the S-enantiomer of ketamine, which has been used for many years as a dissociative anaesthetic (7). In the treatment of depressive disorders, prior to the approval of esketamine, it was only sporadically used in the form of intravenous therapy (8). Esketamine has shown good efficacy and tolerability in large trials (9, 10). The most common adverse reactions in an open-label, long-term extension study with 2769 cumulative patient-years were headache, dizziness, nausea, dissociation, somnolence, and nasopharyngitis (10). However, rare but sometimes serious adverse reactions cannot be fully captured in the context of registration studies because of the conditions that may exclude certain patient populations or include only a limited number of participants. Post-marketing surveillance in the form of spontaneous reporting databases and case reports is of particular importance. In this article, we present two cases of significant creatinine kinase (CK) elevation consistent with rhabdomyloysis after nasal esketamine administration. Rhabdomyolysis is a complex process in which striated muscle is destroyed or damaged. The destruction of muscle tissue results in elevated myoglobin, creatinine kinase, lactate dehydrogenase and electrolyte disturbances due to the release of intracellular components (11). There is no standard definition for the presence of rhabdomyolysis, but in addition to the classic triad of weakness, myoglobinuria and myalgia, which is fully developed in about 10% of cases, there are a number of laboratory parameters for diagnosis (12). Besides myoglobinuria, the determination of creatinine kinase is of particular importance. It has to be considered that the determination of myoglobin in serum is often falsely negative due to its short half-life. There is no established cutoff value for CK; however, a value above five times the upper reference range is often utilized (11, 12). Rhabdomyolysis is often caused by drugs, both illicit and prescribed, alcohol, or other exogenous toxins (13). In addition to underlying myopathy or muscle metabolic defects, neuroleptic malignant syndrome or multifactorial causes are often found in patients with rhabdomyolysis (13). Regardless of the triggering factor and whether there is direct or indirect muscle damage, the common endpoint is the rapid breakdown of damaged skeletal muscle. The electrolyte balance of the muscle tissue is disrupted as a result of direct muscle injury or metabolic disturbances that impair the energy supply. This disruption can lead to membrane damage, which in turn can cause cell necrosis. A further review of this topic may be found in Torres et al. (11). After we have shown the clinical benefits and most prevalent adverse effects of esketamine for the treatment of major depressive disorder, we now present two illustrative cases from our clinical practice. This case series illustrates the occurrence of significant clinical episodes of rhabdomyolysis, underscoring the necessity for heightened vigilance in monitoring for adverse effects when administering esketamine treatment.

## Case presentation case 1

The 33-year-old male patient was admitted to our closed ward as an emergency case for a known recurrent depressive disorder and a current severe episode with acute suicidal tendencies. The patient smoked about 10 cigarettes per day and had rarely consumed alcohol two weeks prior to admission. With regard to illicit substances, there was regular use of cannabis. A drug screen was positive for cannabis, but negative for other substances. There were no known physical illnesses, and there was no history of psychiatric disorders in the family. Pharmacological treatment was started with the serotonin reuptake inhibitor escitalopram (starting dose 5 mg, maximum dose 15 mg) and mirtazapine, a noradrenergic and specific serotonergic antidepressant (starting dose 15 mg, maximum dose 15 mg). If necessary, lorazepam was also administered at a maximum daily dose of 2 mg.

**Figure 1:**
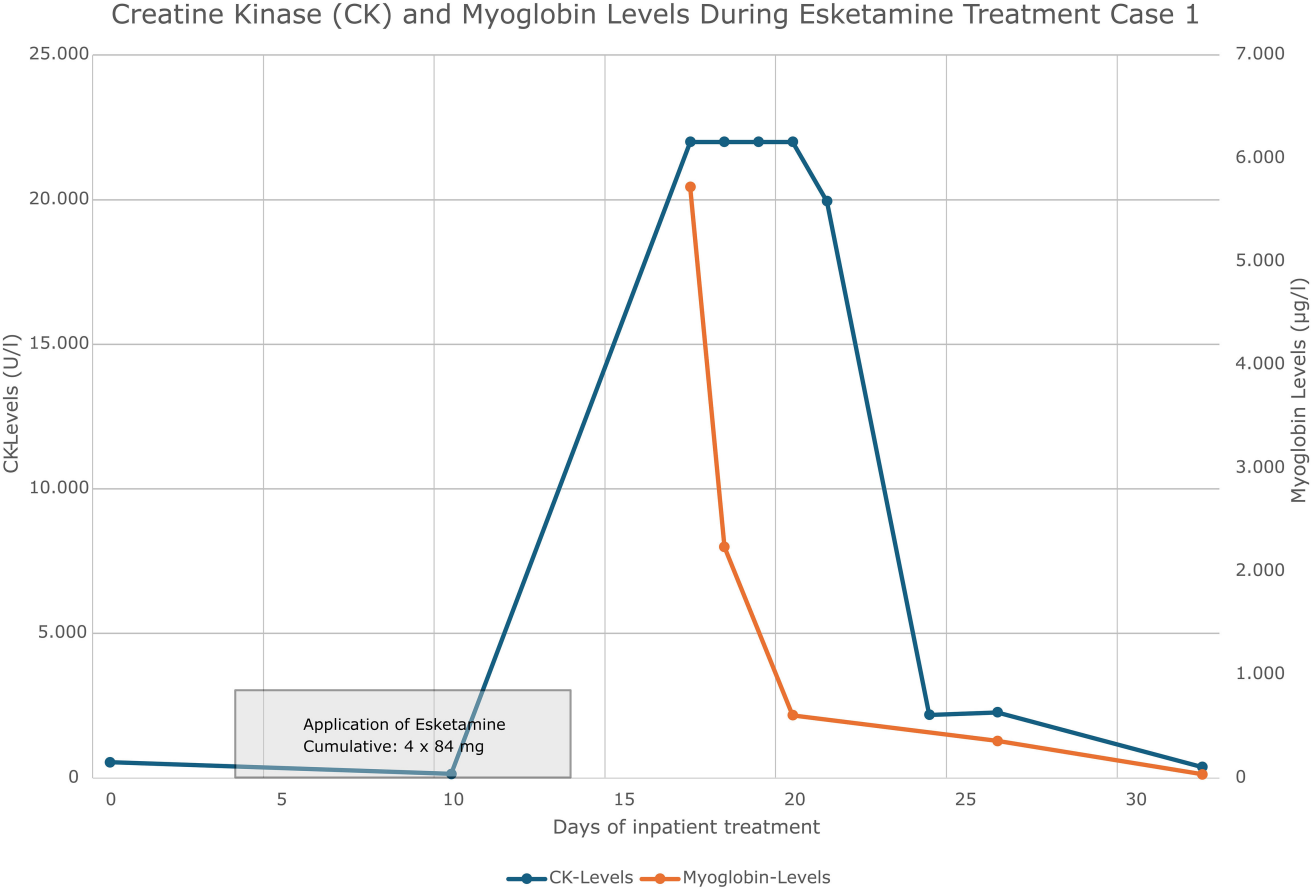
Creatine kinase and myoglobin concentration curve case 1.

The patient quickly distanced himself from acute suicidal intentions and was transferred to the open ward during the inpatient stay. The patient took part in the multimodal therapy concept including sports therapy with low (-to-moderate) exercise intensity. On the fourth day of inpatient treatment after admission, Esketamine 84 mg was started as part of an emergency treatment with still severe depressive symptoms. Intranasal esketamine was administered twice a week at a dose of 84 mg. On day 17, three days after the fourth dose of esketamine, the patient reported fatigue and muscle pain, and a chemical laboratory test was performed. This revealed rhabdomyolysis with significantly elevated CK, CK-myoglobin (CK-MB) and myoglobin levels. The development of creatine kinase and myoglobin can be seen in **Figure 1**. At >22000 U/l, the measured CK levels were above the maximum measurable range of our laboratory; the maximum value for myoglobin was 5722 µg/l. There was also an increase in transaminases and gamma-GT (AST 821 U/l, ALT 208 U/l, and GGT 158 U/l).

### Therapeutic intervention case 1

After suspicion of drug-induced rhabdomyolysis, treatment with esketamine was discontinued, while antidepressant medication with escitalopram and mirtazapine was maintained. The patient was encouraged to have adequate fluid intake and physical activity was reduced. Laboratory parameters were closely monitored. These measures resulted in a decrease in CK and myoglobin (see **Figure 1**). The initial pathological levels of transaminases also decreased and returned to normal. We evaluated the increase in transaminases diagnostically in the context of rhabdomyolysis. Psychopathologically, the patient’s mood remained stable, although his drive was reduced. Forced diuresis or intravenous fluid administration was not necessary because of normal renal values.

### Outcome and follow-up case 1

At the end of inpatient treatment, CK was significantly reduced (355 U/l) and myoglobin was normal. The major depressive episode remitted under antidepressant treatment.

### Patient’s perspective case 1

The patient reported that he had experienced slight muscle soreness following moderate exercise as part of the inpatient therapy concept. He did not report any other complaints. The soreness in the muscles subsided after a few days, and the patient was then asymptomatic.

## Case presentation patient 2

The 22-year-old male patient was admitted for inpatient care following a suicide attempt. Subsequently, the patient was transferred to the closed ward of the psychiatric unit. A severe depressive episode with acute suicidality was diagnosed. This marked the second depressive episode for the patient. The patient was a non-smoker. However, he admitted to using cannabis weekly, the last time being the day before admission. Regular alcohol consumption was denied. No significant medical history was noted, and there were no known familial psychiatric disorders. An antidepressant medication with the serotonin reuptake inhibitor sertraline (starting dose 50 mg, maximum dose 200 mg) and mirtazapine (starting dose 15 mg, maximum dose 30 mg) was initiated.

**Figure 2:**
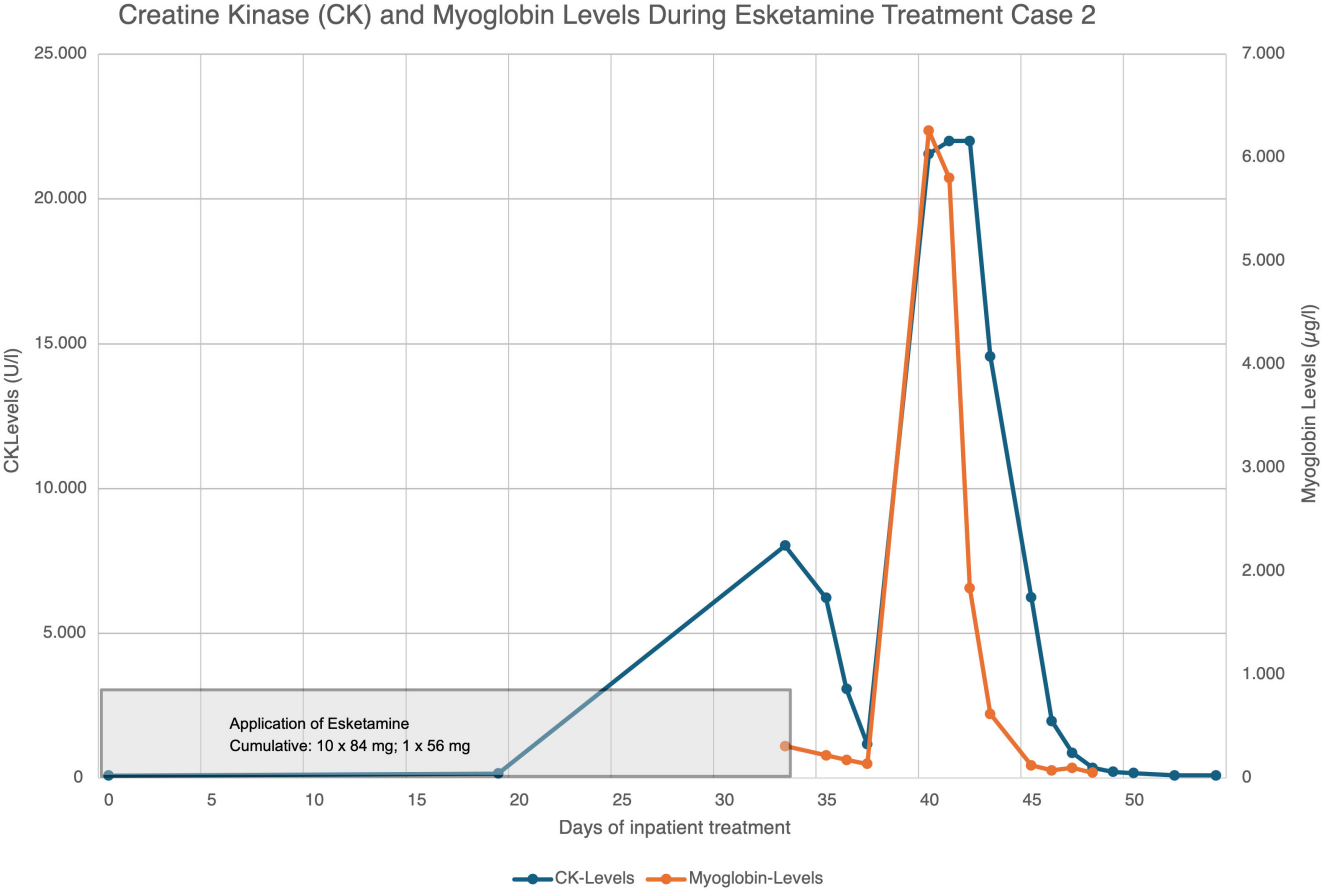
Creatine kinase and myoglobin concentration curve case 2.

The patient’s admission was considered a psychiatric emergency and therefore intranasal esketamine therapy was started on the day of admission at a dose of 84 mg. The patient was quickly able to distance himself from suicidal tendencies and was transferred to our open ward. In total, the patient received 11 doses of esketamine in 5 weeks. The medication with sertraline was taken continuously during the esketamine treatment. Mirtazapine was stopped in week 5. The patient took part in the multimodal therapy concept including sports therapy with low (-to moderate) exercise intensity as part of the inpatient therapy concept. Following the 10th administration of esketamine, the patient reported muscle weakness, and laboratory tests revealed elevated levels of CK with 8032 U/l, myoglobin 310 µg/l and transaminases (AST 194 U/l and ALT 90 U/l). Thus, the next dose of esketamine was reduced with only 56 mg. After this application laboratory tests revealed again markedly elevated levels of CK, CK-MB, and myoglobin and esketamine therapy was discontinued. Further close laboratory checks revealed increasingly rising CK, CK-MB, and myoglobin values, as well as poorer kidney values (Cystatin C 0,98 – 1,12 mg/l) consistent with rhabdomyolysis (see **Figure 2**). Also aspartate transaminase (AST) levels were markedly elevated with maximum levels at 1425 U/l, and alanine transaminase (ALT) at 349 U/l one week after the last esketamine application.

### Therapeutic intervention case 2

After the 10th administration of esketamine and the rising CK, CK-MB, myoglobin and liver values the dose was reduced from 84 mg to 56 mg. When CK, CK-MB, myoglobin and liver values continued to rise even after the application of the reduced dose, treatment with esketamine was discontinued. After discontinuation of the esketamine treatment, the abnormal parameters were monitored closely (initially daily and later at least twice a week). It was noticed that the values continued to rise. 1 week after the end of the esketamine applications, the CK values were no longer in a measurable range (22,000 IU/l). 10 days after the end of esketamine therapy, the values began to fall. 16 days after the end of therapy, CK, CK-MB and myoglobin were in the normal range. The liver values had also fallen significantly with only a minimal transaminase increase of 52U/l of AST 16 days after ending esketamine therapy (see **Figure 2**). The day after the last administration of esketamine, the patient received 1 l of Jonosteril intravenously and 20 mg of furosemide. The patient was encouraged to drink more on a regular basis. The antidepressant treatment with sertraline was temporarily paused in the 7th week of treatment (7 days after the end of es ketamine therapy) and restarted in the 10th week of treatment, up to a maximum dose of 200 mg. During the remainder of his inpatient stay, the patient regularly participated in the multimodal therapy concept, which included psychopharmacotherapy, psychotherapy, and music, exercise, and sports therapy. After moderate exercise, there was a slight increase in CK (max. 7654 U/l), CK-MB (max. 7.33 µg/l), myoglobin (max. 483 µg/l), liver enzymes (max. AST 127 and ALT 61 U/l) and cystatin-C (max. 1.12 mg/l) in weeks 10, 14 and 16. A few days after exercise, all laboratory parameters returned to normal. The patient was hospitalized for a total of 18 weeks. On discharge, the laboratory values were normal except for cystatin-C at 1.12 mg/dl.

### Outcome and follow-up case 2

On discharge, the depressive syndrome was completely remitted and CK, CK-Mb, myoglobin and liver enzymes were normal. At follow-up 2 months after the 18-week inpatient treatment, the patient was still in remission and all laboratory values were normal.

### Patient’s perspective case 2

The patient reported that the symptoms he had experienced were relatively mild. He experienced a musculoskeletal discomfort that lasted approximately two to three days. However, he did not attach any particular significance to these mild symptoms. In the following consultations, he reported feeling well.

After the detailed review of these cases, in which both patients developed rhabdomyolysis shortly after treatment with esketamine, we discuss the potential mechanisms and wider implications of such adverse drug reactions. In consideration of the increasing use of esketamine in treatment-resistant depression this discussion is of particular importance.

## Discussion

We presented two cases, both young male patients with no known physical illness. In both cases, esketamine intrasal was used in a psychiatric emergency, and both patients developed massively elevated CK levels consistent with rhabdomyolysis after a series of esketamine administrations, which decreased after esketamine was discontinued. A causal role of esketamine in the development of rhabdomyolysis should be classified as “possible” or even “probable/likely” according to the criteria of the World Health Organisation (WHO) Drug Monitoring Centre (14), the definitions of Edwards and Aronson (15) and the Naranjo algorithm (16). Accompanying physical activity as part of sports therapy may be an additional factor in the development of rhabdomyolysis. However, it is unlikely to be the sole causal factor; the known case reports of exercise-induced rhabdomyolysis report cases with significantly higher exercise intensities (17). To our knowledge, concomitant risk factors such as high temperature, previous electrolyte disturbances, influenza infection, nutritional problems or creatine intake did not play a role in the reported cases. A risk factor in both cases was male sex, as the available literature suggests that men are at higher risk of rhabdomyolysis (17). Another contributing factor may be the oral comedication. In both cases, antidepressant oral medication with sertraline and mirtazapine was administered. For SSRIs, we found a few case reports of rhabdomyolysis (18–20). Furthermore, there exists an analysis of spontaneous reporting data In which signals for several SSRIs and rhabdomyolysis (21). However, it should be noted that signals do not prove causality and should only be used to generate hypotheses. Since for example in Germany the additional prescription of an SSRI is a prerequisite for the prescription of esketamine nasal for treatment-resistant depression, it is difficult to analyze the influence of oral medication from clinical data. There are also case reports for mirtazapine that describe an association between the development of rhabdomyolysis and oral medication (22–24). In addition, reports suggesting such an association can be found in spontaneous reporting databases (25). However, in the case report presented here, CK remained stable even after resumption of oral medication with sertraline in both cases.

Given that exogenous toxins are a common cause of rhabdomyolysis, the use of cannabis in case 1 should also be discussed. It should be noted that the consumption was not initiated shortly before the event occurred, thus there is no direct temporal connection. The data on rhabdomyolysis caused by herbal cannabis use is ambiguous, but there are some reports and indications that synthetic cannabinoids in particular can be a triggering factor for rhabdomyolysis (26, 27). Nevertheless, cannabis appears to be a substance that can be associated with rhabdomyolysis as a study by Waldman et al. found recently (28).

We do not currently know of any esketamine-induced rhabdomyolysis in the literature. The literature that was found on rhabomyolysis with ketamine is presented below, although it should be noted that the side effect profile is not readily comparable. The literature on ketamine is more extensive as in addition to the new approval of esketamine for the treatment of major depression, ketamine has been used for many years as a dissociative anaesthetic (7). There is also a potential for abuse as a recreational party drug. For the latter, a case series by Weiner et al. described emergency department presentations, two of which resulted in rhabdomyolysis (29). However, both reported cases developed only mild rhabdomyolysis and were very agitated due to intoxication and had to be sedated with benzodiazepines (29). For phencyclidine, a structurally related substance mainly known for recreational abuse, there are several case reports of rhabdomyolysis in acute intoxication (30–32). The mechanism of rhabdomyolysis under phencyclidine appears to be most likely due to increased muscular activity, which was not observed in the studied cases. Other possible mechanisms include metabolic disorders, autoimmune-mediated effects, or membrane effects (33, 34).

It should be noted that the dosages differ significantly between nasal use for depression and misuse or use as an anaesthetic. Nevertheless, the pathomechanisms for the development of muscle damage may be similar.

The present study is a case series showing a close temporal correlation between the use of esketamine and the occurrence of severe rhabdomyolysis in clinical practice. This does not prove a causal relationship, but it does warrant special attention and caution, and should lead to further investigation of the issue in further studies and analysis of spontaneous reporting databases. In everyday clinical practice, when using esketamine, it is advisable to consider and look out for possible symptoms of rhabdomyolysis, such as muscle pain, weakness or a change in the color of the urine, and to carry out laboratory tests, especially of the CK.

## Data availability statement

The original contributions presented in the study are included in the article/supplementary material. Further inquiries can be directed to the corresponding author.

## Ethics statement

Written informed consent was obtained from the individual(s) for the publication of any potentially identifiable images or data included in this article.

## Author contributions

RZ: Conceptualization, Methodology, Validation, Visualization, Writing – original draft, Writing – review & editing. MS: Writing – original draft, Writing – review & editing. BC: Resources, Supervision, Validation, Writing – original draft, Writing – review & editing. KM: Conceptualization, Project administration, Supervision, Validation, Writing – original draft, Writing – review & editing.
